# Structure and Properties of a Natural Competence-Associated Pilin Suggest a Unique Pilus Tip-Associated DNA Receptor

**DOI:** 10.1128/mBio.00614-19

**Published:** 2019-06-11

**Authors:** Mohd Zulkifli Salleh, Vijaykumar Karuppiah, Matthew Snee, Angela Thistlethwaite, Colin W. Levy, David Knight, Jeremy P. Derrick

**Affiliations:** a Lydia Becker Institute of Immunology and Inflammation, School of Biological Sciences, Faculty of Biology, Medicine and Health, Manchester Academic Health Science Centre, The University of Manchester, Manchester, United Kingdom; b Manchester Institute of Biotechnology, The University of Manchester, Manchester, United Kingdom; Duke University School of Medicine

**Keywords:** X-ray crystallography, genetic competence, natural transformation systems, pilus assembly, surface receptor

## Abstract

Natural competence is the term used to describe the uptake of “naked” extracellular DNA by bacteria; it plays a significant role in horizontal genetic exchange. It is associated with type IV pili, and specialized competence pili mediate DNA uptake. Here, we show that the crystal structure of a competence-associated protein from Thermus thermophilus, ComZ, consists of a type II secretion pseudopilin-like domain, with a large β-solenoid domain inserted into the β-sheet of the pilin-like fold. ComZ binds with high affinity to another competence-associated pilin, PilA2, which lies adjacent to the *comZ* gene in the genome. The crystal structure of PilA2 revealed a similar type II secretion pseudopilin-like fold, with a small subdomain; docking simulations predicted that PilA2 binds between the pseudopilin-like and β-solenoid domains of ComZ. Electrophoretic shift analysis and DNase protection studies were used to show that ComZ alone and the ComZ/PilA2 complex are able to bind DNA. Protection against reductive dimethylation was used in combination with mass spectrometry and site-directed mutagenesis to identify two lysine residues in ComZ which are involved in DNA binding. They are located between the two domains in ComZ, on the opposite side from the predicted PilA2 binding site. These results suggest a model in which PilA2 assists ComZ in forming the competence pilus tip and DNA binds to the side of the fiber. The results demonstrate how a type IV pilin can be adapted to a specific function by domain insertion and provide the first structural insights into a tip-located competence pilin.

## Abstract (precis)

Thermus thermophilus is a thermophilic bacterium which is capable of natural transformation, the uptake of external DNA with high efficiency. DNA uptake is thought to be mediated by a competence-associated pilus, which binds the DNA substrate and mediates its transfer across the outer membrane and periplasm. Here, we describe the structural and functional analysis of two pilins which are known to be essential for DNA uptake, ComZ and PilA2. ComZ adopts an unusual structure, incorporating a large β-solenoid domain into the pilin structural framework. We argue on structural grounds that this structure cannot readily be accommodated into the competence pilus fiber unless it is at the tip. We also show that ComZ binds DNA and identify two lysine residues which appear to be important for DNA binding. These results suggest a model in which ComZ and PilA2 form a tip-associated DNA receptor which mediates DNA uptake.

## INTRODUCTION

Horizontal gene transfer (HGT) is a process whereby genetic material is transferred between bacterial cells. HGT is mediated by three different mechanisms: conjugation, transduction, and natural transformation (NT); it therefore plays an important role in bacterial adaptive evolution. NT is characterized by the ability of bacteria to take up “naked” DNA from outside the cell: one strand of the DNA molecule is degraded, and the other is transported into the cytoplasm (1–3). DNA uptake and translocation into the cytoplasm is an active process, and DNA taken up in this way can be used as food for genome repair or to generate genetic diversity. The latter property has implicated NT in mediating antimicrobial resistance in several pathogenic bacterial species, such as respiratory tract flora Haemophilus influenzae, Streptococcus pneumoniae, and Neisseria meningitidis. Natural competence is also found in thermophilic, as well as mesophilic, bacteria, notably Thermus thermophilus, where it could have a role in assisting environmental adaptation (4).

Natural competence in T. thermophilus is mediated by a multiprotein complex, consisting of 16 proteins which span the outer membrane, the periplasm, and the inner membrane (4, 5). Competence genes in T. thermophilus are upregulated under stress conditions such as starvation, nutrient deprivation, overpopulation, and even low temperature (6). H. influenzae and *Neisseria* species exhibit a strong preference for extracellular DNA fragments that contain specific uptake sequences (7–9). T. thermophilus, in contrast, can take up DNA without apparent sequence specificity; it is therefore a highly transformable and rapidly adaptable bacterial species, growing in hostile environments, with temperatures ranging from 50 to 82°C and pH values ranging from 6 to 9 (4, 10, 11). DNA uptake in T. thermophilus is a fast and efficient process; estimated uptake velocities of up to 40 kb s^−1^ have been reported (11).

NT in Gram-negative bacteria is connected to the biogenesis of type IVa (T4a) pili (3). T4a pili are long, thin fibers which protrude from the bacterial surface; they are responsible for mediating host cell adhesion and a type of bacterial movement called twitching motility, as well as the uptake of DNA (12). Pilin subunits are exported into the periplasm, cleaved by a dedicated signal peptidase, and assembled into a pilus fiber by a complex of proteins in the cytoplasm and inner membrane. In Gram-negative organisms, this assembly platform comprises three transmembrane proteins, PilN, PilO, and PilC, a soluble protein, PilM, which binds to PilN, and a dedicated ATPase (PilF in T. thermophilus), which catalyzes pilus fiber assembly (6, 13–19). As well as being responsible for pilus formation, these proteins are all required for NT in T. thermophilus (4, 17). In addition, specialized competence-specific proteins are required; recent work in T. thermophilus has shown that a competence-associated protein, ComEA, is associated with the inner membrane and is responsible for binding to DNA (6). Another conserved protein, ComEC, forms a polytopic transmembrane protein and the channel for DNA passage across the inner membrane. Both proteins are highly conserved, with orthologs in Gram-positive as well as Gram-negative bacteria (1, 20).

The process of DNA uptake from the extracellular milieu and subsequent transport into the cytoplasm is divided into several discrete steps, although the details of each stage remain unclear (1, 3, 21). The initial step requires binding of DNA outside the cell, probably to a DNA-specific receptor associated with a type IV pilus. In Gram-negative bacteria, transport needs to be negotiated across the outer membrane; PilQ, a member of the secretin family, is an integral outer membrane protein which provides a channel for passage of type IV pili (22–24). The observation that PilQ is capable of binding DNA suggests a similar role for DNA transport, although details of how this occurs are obscure (25). Once inside the periplasm, the incoming DNA is bound by ComEA and one strand is degraded by an endonuclease. Transport across the inner membrane is mediated by ComEC; the DNA strand is subsequently bound by DprA (26), which recruits RecA into a complex to promote homologous recombination. Many details of this basic model remain unclear, however, notably the identity of the protein initially responsible for DNA binding and the precise mechanism by which uptake is powered. Curiously, mutations to the two retraction-specific PilT ATPases in T. thermophilus do not impair natural competence (19), although other ATPases could be involved in retraction of the competence pilus.

Type IV pili are made up of polymers of noncovalently linked pilin proteins; type IV pilins adopt a canonical structure, consisting of an N-terminal hydrophobic α-helix packed against an antiparallel β-sheet (12, 27). Several structural variations on this theme have been described, including addition of entire domains (28, 29). Pili are mainly composed of a single pilin, but minor pilins, present in much lower quantities, can play a crucial role in pilus formation. For example, the minor pilins *pilHIJK* in N. meningitidis play a role in pilus assembly (30), possibly through modulation of pilus surface density (31, 32). Similar observations have been made in *Pseudomonas*, and minor pilins are known to adopt the type IV pilin canonical fold, with noted similarities to the type II secretion system pseudopilins (33–35). A specialized minor pilin, ComP, from N. meningitidis is required for DNA recognition in natural transformation but is dispensable for T4a pilin formation and other functions (36). ComP has a structure similar to that of other T4a pilins and exhibits a DNA uptake sequence (DUS) binding specificity consistent with it acting as the primary DNA receptor (37). An electropositive strip of surface residues forms a specific docking surface for the DNA ligand, and selectivity toward different DUS variants extends to ComP homologs from other *Neisseria* species (38). However, ComP is apparently confined to *Neisseria* and remains the sole example to date of a T4a pilin which specifically binds to DNA. Moreover, Hepp and Maier report that an N. gonorrhoeae *comP* mutant is not impaired in DNA uptake, suggesting that other components are involved in DNA recognition (39). The question of the identity of the primary DNA recognition pilin therefore remains open in most naturally competent Gram-negative bacteria.

Here, we describe a structural and functional analysis of ComZ, a pilin-like protein which is part of a locus in T. thermophilus HB27 and is known to be essential for NT but not piliation (40). We show that the ComZ structure incorporates both pilin-like and β-solenoid domains, binds specifically to an adjacent minor pilin in this specialized locus, and is able to recognize DNA. Based on these observations, we propose that ComZ functions as a tip pilin and receptor for the initial DNA binding step outside the bacterial cell.

## RESULTS

### ComZ adopts an unusual type IV pilin-like structure.

Previous work on T. thermophilus HB27 by Friedrich et al. identified a locus containing five open reading frames (ORFs) associated with natural competence (40). Four ORFs (*pilA1* to *pilA4* [*pilA1-4*]) were type IVa pilin-like genes, and a fifth, *comZ*, encoded an unusual and much larger protein that lacked some of the sequence features characteristic of pilin genes (12, 35). Mutation of *pilA1-3* and *comZ* leads to a loss of natural competence, although piliation is maintained (40). We set out to express and purify PilA1-3 and ComZ, in each case omitting the first 30 to 33 residues. This is a common strategy used for type IV pilin expression, because it removes the hydrophobic portion of the conserved N-terminal α-helix. ComZ was crystallized and the structure was determined to 2.72-Å resolution (see Table S1 in the supplemental material). The structure had three ComZ molecules in the asymmetric unit, but analysis by the protein interface analysis server PISA (41) did not indicate any significant crystal contacts. Size exclusion chromatography (SEC) during purification was consistent with the recombinant ComZ fragment forming a monomer in solution.

10.1128/mBio.00614-19.8 TABLE S1 X-ray data collection and refinement statistics for ComZ. Download Table S1, DOCX file, 0.01 MB. Copyright © 2019 Salleh et al. 2019 Salleh et al. This content is distributed under the terms of the Creative Commons Attribution 4.0 International license.

**FIG 1:**
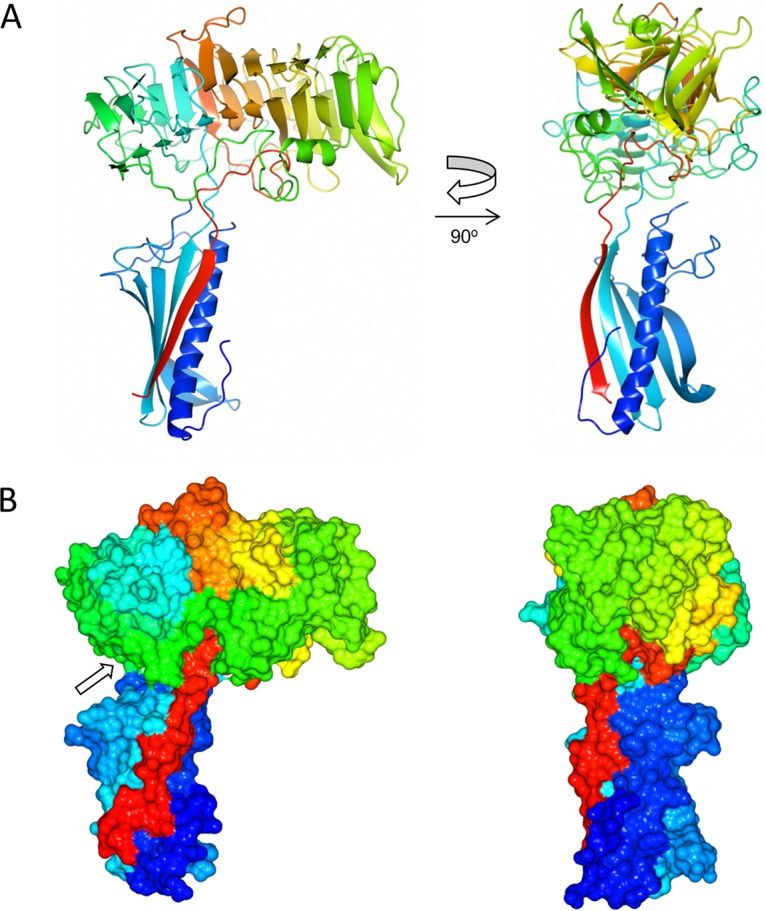
Structure of T. thermophilus ComZ. (A) Two orthogonal ribbon plot views of ComZ, colored on a gradient from the N (blue) to the C (red) terminus. (B) Two surface plots of ComZ, with the same orientations and color scheme as panel A. The arrow indicates the long loop which connects the two ends of the β-helix.

Figure 1 shows the overall structure of ComZ: it forms two clearly identifiable domains. ComZ was not identified as a type IV pilin by PilFind (42), presumably because it does not contain key characteristic sequence motifs; however, the N-terminal domain adopts a type IV pilin-like fold, suggesting that it forms a structural component of a pilus fiber. The second domain forms a complex β-solenoid structure, which is inserted between the penultimate and last β-strands in the pilin fold through two short linkers.

The pilin fold is formed from five antiparallel β-strands packed against a single α-helix, with an extensive loop region between the end of the α-helix (Asn39) and the beginning of the first β-strand (Asn71). From a comparison of the three chains, the relative orientation of the two domains is invariant (Fig. S1). The segmental mobility between the two domains is likely to be constrained by contacts formed from the extensive loop regions, which emanate from the β-solenoid and pilin domains. Most notably, an extensive loop and short α-helix from Glu216 to Ala301 connects the two ends of the β-helix, passing between the two domains and making contact with the second of the two peptide linkers which join both domains (Fig. 1B, arrow). The loop packs against the concave face of the β-solenoid and buries hydrophobic sidechains, contributing to the rigidity of the structure. This lack of flexibility of the β-solenoid domain could play a part in the proposed role of ComZ as a tip receptor for DNA binding (discussed further below).

10.1128/mBio.00614-19.1 FIG S1 Superposition of ComZ chains A, B, and C. Chains B and C were superimposed onto chain A using CCP4MG (72), with root mean square deviations of 1.1 and 0.93 Å, respectively. Chain A is in blue, chain B is in gold, and chain C is in orange. Download FIG S1, DOCX file, 1.0 MB. Copyright © 2019 Salleh et al. 2019 Salleh et al. This content is distributed under the terms of the Creative Commons Attribution 4.0 International license.

### Comparison of ComZ with related structures.

**FIG 2:**
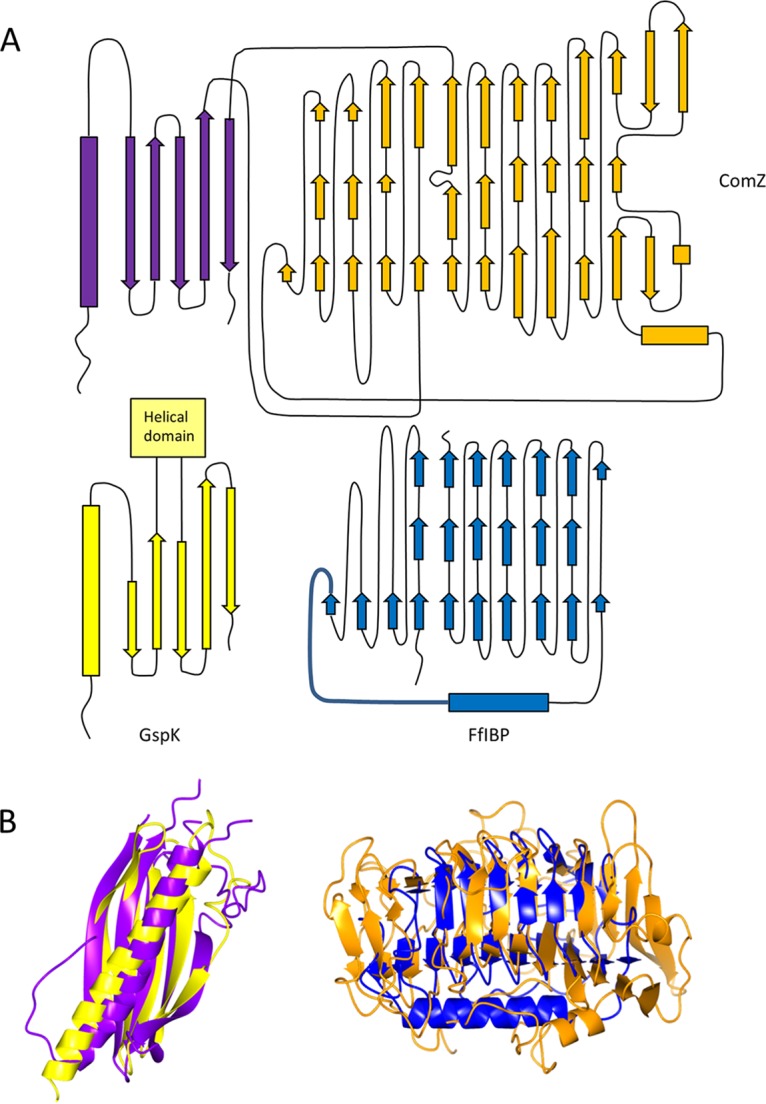
Topology and comparison of ComZ domains with structurally related proteins. (A) Topology of ComZ and related folds. (Upper) Topology diagram of ComZ, with the pilin-like domain in purple and the β-solenoid domain in orange. (Lower) Folds of the related proteins GspK and FfIBP are aligned below their mapped superpositions to the equivalent ComZ domains. (B) Superpositions of related structural folds with their equivalent domains from ComZ. (Left) GspK from E. coli (PDB entry 3CIO; pilin domain only) is shown in yellow, and the ComZ pilin-like domain is in purple. (Right) The ice-binding protein FfIBP (4NU2) from Flavobacterium frigoris is in dark blue, and the β-solenoid domain is in orange. Superpositions were carried out using SSM matching, as implemented in CCP4MG (72); root mean square deviation values were 3.25 Å (GspK) and 2.33 Å (FfIBP). Colors are the same as those for panel A.

The Protein Data Bank now contains an extensive library of type IV pilin and pseudopilin structures (12); the ComZ pilin-like domain was therefore searched for structural homologs using the fold recognition program DALI (43). The closest match was the type II secretion system pseudopilin GspK, which forms the tip of a pseudopilus heterotrimeric complex (GspK/GspI/GspJ) (29). The fold topology of GspK has striking similarities with the ComZ pilin-like domain (Fig. 2A); the principal difference lies in the insertion point of the additional domain in each structure. In the case of ComZ, the insertion lies between the penultimate and last β-strands, whereas in GspK, insertion of an α-helical domain is between the third and fourth β-strands (Fig. 2B, topology diagrams). Our previous work has highlighted the similarity of T. thermophilus minor pilins to type II secretion system pseudopilins (35), but the structure of ComZ also shows how the canonical pilin domain can be adapted by various domain insertions, possibly to add additional functionality. The structure of the minor type IVb pilin CofB from enterotoxigenic Escherichia coli (ETEC) has parallels with ComZ, in that both are structurally related to GspK and both have additional β-rich domains outside the pilin fold (28). The CofB structure consists of an N-terminal pilin domain followed by a short β-repeat section and a β-sandwich domain at the C terminus. Interestingly, the C-terminal regions of CofB are thought to be necessary to initiate type IVb pilus assembly (28).

**FIG 3:**
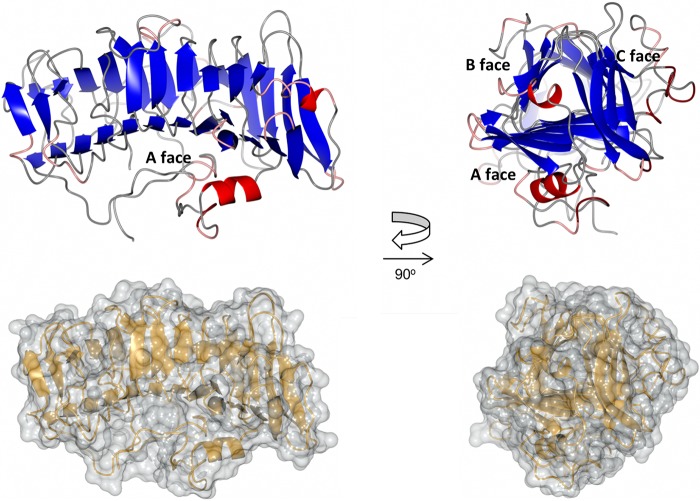
Detail of the β-solenoid domain from ComZ. (Upper) Two orthogonal ribbon plot views of the β-solenoid domain from ComZ, colored by secondary structure: β-strand (blue), α-helix (red), turn (pink), and coil (gray). Faces of the solenoid are labeled on the right. (Lower) Surface models, in the same orientations as that of the upper panel, superimposed on ribbon plot.

The β-solenoid domain comprises ∼380 residues and constitutes the majority of the ComZ structure. It is formed from parallel β-strands arranged in a triangular β-helix, stabilized by intramolecular main-chain hydrogen bonds and a hydrophobic core characteristic of β-solenoid structures. The domain forms an elongated structure, about 60 Å in length, with a triangular cross-section (Fig. 3). There are several irregularities in the fold, notably at the end between residues 301 and 362, where the β-solenoid breaks down and is replaced by two pairs of antiparallel β-strands (Fig. 2B, far right of the topology diagram). Viewed from the side, it is apparent that the β-solenoid has a pronounced bend (Fig. 3). We labeled the faces of each side of the solenoid A, B, and C by analogy with the fold of the ice-binding protein (discussed below). Several long loops emanate from the β-strands and pack against the faces of the β-solenoid; this phenomenon is most pronounced for the A face, where a network of loops and a short α-helix pack against the parallel β-sheet (Fig. 3). Another long loop from Asn182 to Ala208 packs against the C face and a shorter loop, from Thr476 to Lys489, against the B face.

The size of the β-solenoid domain, combined with the rigid orientation of the two domains discussed above, led us to examine whether it could be incorporated into a model for the assembled T4a pilus fiber. Using the structure of the pilus from N. meningitidis (44), we superimposed the ComZ pilin domain: the β-solenoid domain overlaps well with the fourth and fifth pilin subunits in the fiber (Fig. S2). Even with minor adjustment of the relative orientation of the β-solenoid and pilin domains in ComZ, it is unlikely that it could be stably incorporated into the fiber. In fact, the β-solenoid domain is placed in line with the central axis of the fiber, placing it in an ideal orientation to act as a tip adhesin. We acknowledge that other models for T4a pilus fiber differ in their details, but the central packing of N-terminal hydrophobic helices is a common feature; our conclusion is therefore unlikely to be altered substantially by these differences (12).

10.1128/mBio.00614-19.2 FIG S2 Pilin domain from ComZ (chain A) was superimposed onto a single chain from the structure of N. meningitidis pilin (PDB accession code 5KUA) using CCP4MG (72). ComZ A is in red, and the N. meningitidis pilin is in green. Download FIG S2, DOCX file, 2.0 MB. Copyright © 2019 Salleh et al. 2019 Salleh et al. This content is distributed under the terms of the Creative Commons Attribution 4.0 International license.

A fold recognition search using DALI (43) identified an ice-binding protein, FfIBP from Flavobacterium frigoris (PDB accession code 4NU2), as the closest structural relative of the ComZ β-solenoid domain (45). The topology of the core fold of the two proteins is similar, covering 10 turns of the solenoid (Fig. 2B). However, there are several key differences between the two structures. The ComZ solenoid is longer, more irregular, and with many more extensive loops. FfIBP has an α-helix which packs against the A face, performing a structural role similar to that of the loops in the ComZ structure (Fig. 2A, right). FfIBP is a member of a class of ice-binding proteins, which are characterized by their β-solenoid folds (46). The ice-binding residues in FfIBP have been mapped to face B (45), but we found no obvious sequence conservation in the equivalent positions for ComZ. Given that the structural conservation of ComZ with this class of ice-binding proteins did not seem to provide any insight into its function, we sought to investigate the properties of ComZ in other ways.

### Structure of the competence-associated type IV pilin PilA2.

Three competence-associated pilin genes, *pilA1-pilA2-pilA3*, are found adjacent to *comZ* in the T. thermophilus strain HB27 genome (11). Unlike ComZ, each ORF was positively identified as a type IV pilin by PilFind (42). Recently we described a strategy for systematic expression and characterization of pilins from T. thermophilus HB8, which involved removal of the signal sequence and part of the N-terminal helix, and incorporation of a purification tag (35). We applied the same strategy to PilA1, PilA2, and PilA3 from the HB27 strain and purified each truncated pilin to homogeneity. PilA2 gave crystals with good diffraction qualities, and the structure was determined to 1.39-Å resolution; it forms a distinctly identifiable domain with a small subdomain insertion (Table S2 and Fig. S3A). PilA2 adopts a type IV pilin fold, a single α-helix packed against four antiparallel β-strands, with a small subdomain inserted between the end of the first α-helix and the beginning of the fourth β-strand, consisting of three short antiparallel β-strands and a short α-helix, linked by an extensive loop region. A search with DALI (47) identified close structural homology with the type II secretion system pseudopilins EpsI from Vibrio vulnificus (48) and GspI from E. coli (29), as well as the minor type IV pilin TTHA1218 from T. thermophilus HB8 (35). Although all three related pilin structures share the same fold, the subdomain is consistently absent (Fig. S3B).

10.1128/mBio.00614-19.3 FIG S3 Structure of the competence-associated type IV pilin PilA2. (A) Two orthogonal ribbon plot views of PilA2, colored by secondary structure. (B, upper) Topology diagram for PilA2. The additional subdomain is indicated by the blue box; (middle) topology diagrams for EpsI from V. cholerae, GspI from E. coli, and TTHA1218 from T. thermophilus; (lower) superposition of PilA2 structure (red) with EpsI (blue), GspI (green), and TTHA1218 (yellow). See the text for other details. Download FIG S3, DOCX file, 0.4 MB. Copyright © 2019 Salleh et al. 2019 Salleh et al. This content is distributed under the terms of the Creative Commons Attribution 4.0 International license.

10.1128/mBio.00614-19.9 TABLE S2 X-ray data collection and refinement statistics for PilA2. Download Table S2, DOCX file, 0.01 MB. Copyright © 2019 Salleh et al. 2019 Salleh et al. This content is distributed under the terms of the Creative Commons Attribution 4.0 International license.

### ComZ binds specifically to the competence-associated pilin PilA2.

**FIG 4:**
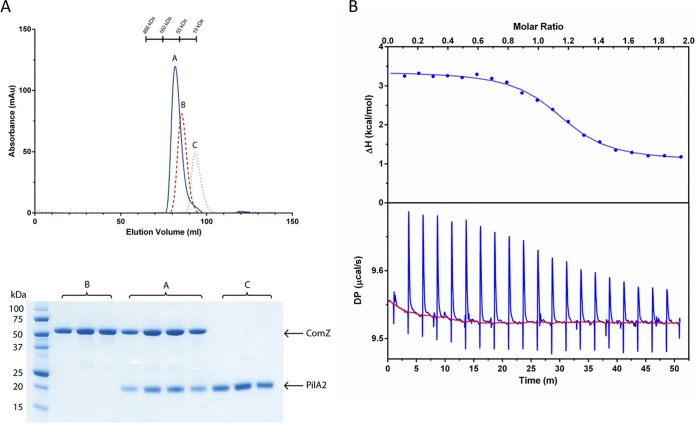
Binding of PilA2 to ComZ. (A) Size exclusion chromatography analysis. (Upper) Elution profiles (absorption at 280 nm) of ComZ-PilA2 mixture (profile A), ComZ alone (profile B), and PilA2 alone (profile C). Separation was carried out on a HiLoad 16/600 Superdex 200 PG column (GE Healthcare), with a flow rate of 1 ml/min in 25 mM Tris-HCl, pH 8.0, 200 mM NaCl, 5% glycerol. ComZ (0.036 μmol) and PilA2 (0.023 μmol) were loaded. (Lower) SDS-PAGE of eluted peaks from each SEC run. (B) Titration of PilA2 into ComZ using isothermal titration calorimetry (ITC). Two-μl aliquots of recombinant PilA2 (314 μM) were titrated into the sample cell containing 300 μl of ComZ (33 μM). (Lower) Raw data with fitted baseline. (Upper) Data fitted assuming a single binding site. Fitted parameters are the following: stoichiometry (*n*), 1.1; equilibrium binding constant (*K_d_*), 0.69 μM; enthalpy change (Δ*H*), 2.3 kcal/mol; Δ*G* = −8.4 kcal/mol; *T*Δ*S* = 10.7 kcal/mol.

Given the structural similarity of the ComZ pilin domain to GspK, we reasoned that ComZ should bind to at least one other pilin, if it is indeed incorporated into a pilus structure. Size exclusion chromatography (SEC) profiles for ComZ and PilA2, ComZ alone, and PilA2 alone are shown in Fig. 4A. The ComZ/PilA2 complex eluted in a single peak (∼80 kDa), at a higher apparent mass than ComZ alone (∼60 kDa); the complex peak contained both proteins (Fig. 4A, lower). Specific binding of the two proteins was confirmed by isothermal titration calorimetry (ITC), which gave a stoichiometry of 1.1 and an equilibrium binding constant (*K_d_*) of 0.69 μM (Fig. 4B).

Similar studies of binding to ComZ were carried out using the PilA1 and PilA3 pilins. PilA1 failed to show any evidence of binding to ComZ (Fig. S4A). The elution profile of PilA3 from the SEC column suggested the formation of oligomers, so it was not possible to use this method to determine binding to ComZ (Fig. S4B). Experiments were also conducted where ComZ was preincubated with PilA2 and either PilA1 or PilA3, before separation by SEC, to examine whether PilA1 or PilA3 affects the binding of PilA2 to ComZ indirectly. The results suggested that this was not the case, however, and that the presence of PilA1 or PilA3 had no discernible effect on the elution profile of the ComZ/PilA2 complex (Fig. S5A and B). We sought to confirm and extend these observations using an affinity tag assay. ComZ was incubated with PilA2 or PilA3, each of which contains a Strep-tag; the mixture was then passed through a Strep affinity column, unbound protein was eluted, the column was washed, and eluted protein was analyzed by SDS-PAGE. ComZ coeluted with PilA2 (Fig. S6A) but not with PilA3 (Fig. S6B). The reverse experiment, which used a Ni affinity column to bind ComZ, showed that PilA2, but not PilA3, coeluted with ComZ (Fig. S6C). We conclude that ComZ selectively binds to PilA2, but not PilA1 or PilA3.

10.1128/mBio.00614-19.4 FIG S4 Size exclusion chromatography analysis of ComZ interaction with PilA1 and PilA3. (A) ComZ and PilA1. (Upper) Elution profiles (absorption at 280 nm) of ComZ-PilA1 mixture (profiles B and C), ComZ alone (profile A), and PilA1 alone (profile D). Separation was carried out on a HiLoad 16/600 Superdex 200 PG column (GE Healthcare) at a flow rate of 1 ml/min in buffer of 25 mM Tris-HCl, pH 8.0, 200 mM NaCl, 5% glycerol. Prior to SEC separation, the proteins were incubated in the buffer at 4°C for 30 min. Aliquots of 0.036 μmol of ComZ and 0.027 μmol of PilA1 were loaded. (Lower) SDS-PAGE of eluted peaks from each SEC run. (B) ComZ and PilA3. (Upper) Elution profiles (absorption at 280 nm) of ComZ-PilA3 mixture (profile A), ComZ alone (profile B), and PilA3 alone (profile C). SEC separation was carried out as for panel A. Download FIG S4, DOCX file, 0.6 MB. Copyright © 2019 Salleh et al. 2019 Salleh et al. This content is distributed under the terms of the Creative Commons Attribution 4.0 International license.

10.1128/mBio.00614-19.5 FIG S5 Size exclusion chromatography analysis of ComZ/PilA2 interaction with PilA1 and PilA3. (A) ComZ/PilA2 and PilA1. (Upper) Elution profiles (absorption at 280 nm) of ComZ-PilA2-PilA1 mixture (profile A and D), ComZ alone (profile B), PilA2 alone (profile C), and PilA1 alone (profile E). Separation was carried out on a HiLoad 16/600 Superdex 200 PG column (GE Healthcare), with a flow rate of 1 ml/min in buffer containing 25 mM Tris-HCl, pH 8.0, 200 mM NaCl, 5% glycerol. Prior to SEC separation, the proteins were incubated in the buffer at 4°C for 30 min. Aliquots of 0.036 μmol of ComZ/PilA2 and 0.027 μmol of PilA1 were loaded. (Lower) SDS-PAGE of eluted peaks from each SEC run. (B) ComZ/PilA2 and PilA3. (Upper) Elution profiles (absorption at 280 nm) of ComZ-PilA2-PilA3 mixture (profile B), ComZ alone (profile A), PilA2 alone (profile C), and PilA3 alone (profile D). SEC separation was carried out as described for panel A. (Lower) SDS-PAGE of eluted peaks from each SEC run. Download FIG S5, DOCX file, 0.5 MB. Copyright © 2019 Salleh et al. 2019 Salleh et al. This content is distributed under the terms of the Creative Commons Attribution 4.0 International license.

10.1128/mBio.00614-19.6 FIG S6 ComZ affinity binding assays. (A) ComZ/PilA2. Both proteins were incubated in 25mM Tris-HCl, pH 8.0, 200 mM NaCl, 5% glycerol at 4°C for about 30 min and applied to a Streptavidin column, and eluted proteins were examined by SDS-PAGE. Three mg of ComZ and 2 mg of PilA2 were loaded. (B) ComZ/PilA3. Both proteins were incubated together and applied to a Streptavidin column, and eluted proteins examined by SDS-PAGE. Three mg of ComZ and 2 mg of PilA3 were loaded. Buffer and temperature were as described for panel A. (C) ComZ/PilA2/PilA3. Both proteins were incubated together and applied to a Ni affinity column, and eluted proteins were examined by SDS-PAGE. Buffer and temperature were as described for panel A. Download FIG S6, DOCX file, 0.8 MB. Copyright © 2019 Salleh et al. 2019 Salleh et al. This content is distributed under the terms of the Creative Commons Attribution 4.0 International license.

### Characterization of ComZ binding to dsDNA.

**FIG 5:**
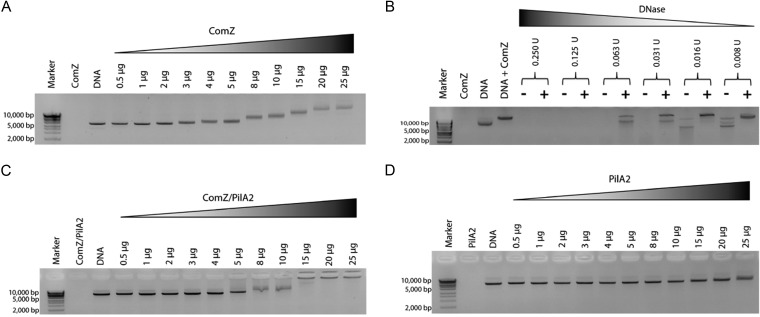
Binding of ComZ to dsDNA. (A) ComZ-dsDNA interaction studied by EMSA. Increasing quantities of ComZ were added to 200 ng of linear dsDNA, as indicated. The ComZ lane is a control without DNA, and the adjacent lane, labeled DNA, is without added ComZ. (B) Protection of DNase digestion by ComZ. The digestion of linear dsDNA by various quantities of DNase was compared in the presence and absence of 8.7 μM ComZ. (C) ComZ/PilA2-dsDNA interaction studied by EMSA. Other conditions were as indicated for panel A. (D) PilA2-dsDNA interaction studied by EMSA. Other conditions were as indicated for panel A. DNA was detected using SafeView nucleic acid stain (NBS Biologicals). Marker used was HyperLadder 1 kb (Bioline).

It was previously reported that *comZ*, *pilA1*, *pilA2*, and *pilA3 Thermus* mutants are impaired in natural competence but retain type IV pili (3). Given that the structure of ComZ suggested it functions as a tip pilin, we examined whether it was able to bind to DNA. Studies by electrophoretic mobility shift assay (EMSA) showed that increasing quantities of ComZ reduce double-stranded DNA (dsDNA) migration (Fig. 5A). DNA binding proteins which recognize specific sites can produce smears on EMSA when tested against nonspecific DNA. In the case of ComZ, we observe discrete DNA bands. We suggest that this is explained by binding of multiple molecules of ComZ, gradually reducing the mobility of each DNA strand. This explanation is consistent with the fact that T. thermophilus does not exhibit any DNA sequence preference (4), unlike *Neisseria* spp. (37), for example. We sought to verify this conclusion by conducting a DNase protection experiment: increasing quantities of DNase I were added to ComZ and dsDNA (Fig. 5B). ComZ was able to inhibit nonspecific hydrolysis of DNA by DNase, effectively providing protection at concentrations where substantial degradation occurred. The results are consistent with multiple copies of ComZ binding to each dsDNA duplex in a non-sequence-dependent manner. In the presence of PilA2, the ComZ/PilA2 complex reduced DNA mobility to a greater extent than ComZ alone, indicative of the higher mass of the ComZ/PilA2 complex (Fig. 5C). Virtually no reduction in DNA mobility was recorded in the presence of PilA2 alone (Fig. 5D).

**FIG 6:**
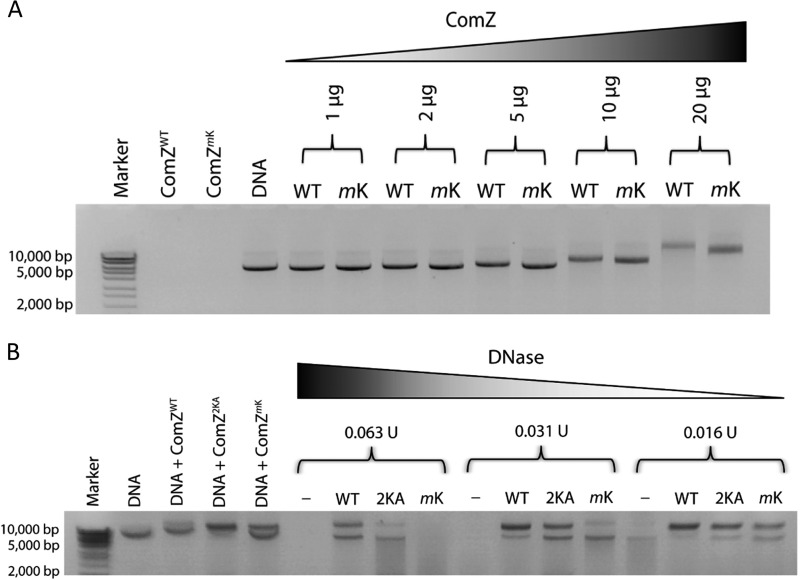
Inhibition of DNA binding by dimethylated ComZ. (A) Increasing quantities of unmodified (WT) and dimethylated ComZ (*m*K), as indicated, were added to 200 ng of linear dsDNA. (B) DNase digestion protection was examined by comparison of the degradation of linear dsDNA in the absence of ComZ and presence of unmodified wild-type ComZ, dimethylated K98A/K233A ComZ (2KA), and dimethylated wild-type ComZ (*m*K). Marker used was HyperLadder 1 kb (Bioline).

**FIG 7:**
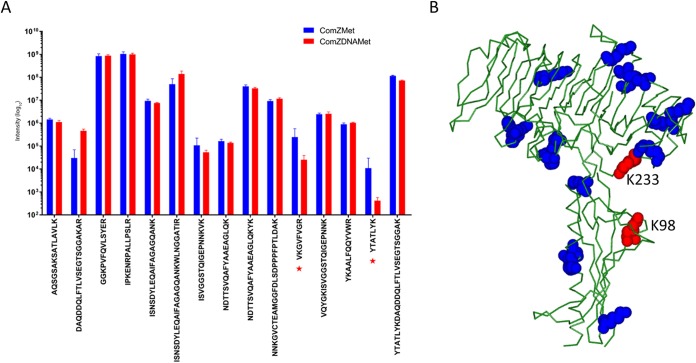
LC-MS analysis of ComZ-DNA binding. (A) Intensities of dimethylated peptides in the absence (blue) and presence (red) of dsDNA (*n* = 3; values are ± standard errors from the means). (B) Location of lysine residues in ComZ. The two protected lysines, K98 and K233, are shown in red, and other lysines are in blue.

In order to map the DNA binding site on ComZ, we developed a method based on the protection of lysine residues from reductive dimethylation by bound DNA. We reasoned that lysines were likely to be involved in DNA recognition, and inspection showed that they cover both ComZ domains. Protocols for reductive dimethylation are well established and used to assist in crystallization (49). Dimethylation of ComZ impaired its ability to bind to DNA, as measured by EMSA (Fig. 6A). ComZ was exposed to the dimethylation reagent in the presence and absence of DNA and digested with protease, and peptides were identified from analysis by liquid chromatography-mass spectrometry (LC-MS) (Fig. 7A). Two peptides showed a significant reduction in intensity due to DNA protection and were identified as originating from modification of lysines 98 and 233. We noted that K98 and K233 are located between the two domains in ComZ, on the side of the L-shaped structure (Fig. 7B). To provide independent verification for this observation, both lysines were mutated to alanine, and the ability of the ComZ K98A/K233A double mutant to protect DNA from DNase digestion was compared to that of wild-type (WT) and dimethylated ComZ (Fig. 6B). Dimethylation had a substantial impact on the ability of ComZ to protect against degradation; the K98A/K233A mutant was also significantly impaired compared to the wild type, providing additional evidence that these lysine residues are indeed involved in DNA binding, although other lysines, and indeed other residues, are likely involved.

### Modelling of the ComZ-PilA2-DNA complex.

**FIG 8:**
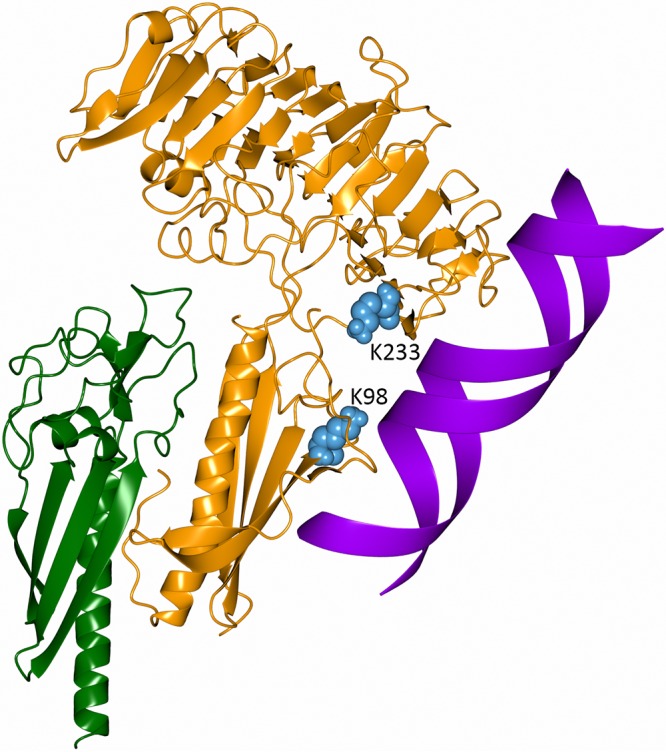
Model for the ComZ-PilA2-DNA complex. PilA2 is shown in green, ComZ in orange, and DNA in purple. See the text for further details. The figure was generated using CCP4MG (72).

With structures of ComZ and PilA2 and some knowledge of the location of the DNA binding site on ComZ, we set out to model the tripartite complex. We reasoned that the complex between ComZ and PilA2 was likely formed by interaction between the two N-terminal helices in each structure, which is a consistent feature in the structures of assembled type IV pili. Using the complex of GspIJK as a starting model (29), the ComZ pilin domain was superposed onto GspK and PilA2 onto GspI, which is a close structural homolog. The model was adjusted to remove steric clashes, and six starting models were generated by rotation about the PilA2 helix, approximately 25° apart. Each model was sampled with 1,000 independent docking simulations implemented in ROSETTA (50), and the lowest-energy model was selected from the total of 6,000. This model places PilA2 such that its subdomain fits in the hinge region between the pilin and solenoid domains of ComZ and on the opposite side from K98 and K233 (Fig. 8). To generate a model for the ComZ-DNA complex, a 21-base B-form duplex DNA structure was generated using the make-na server (51) and docking carried out using HADDOCK2.2 (52), with the constraints that K98 and K233 were selected as active residues. The resulting model predicted contacts between the DNA and both domains in ComZ (Fig. 8). In particular, this arrangement shows DNA binding to residues 250 to 255 at the end of the β-solenoid and part of the extensive loop region, which links the pilin α-helix with the first β-strand.

## DISCUSSION

The initial encounter of DNA by a specific receptor outside the cell is an essential first step in the DNA uptake by NT in Gram-negative bacteria. Although this outline model is consistent across different naturally competent Gram-negative organisms, details vary. For example, some species have specificity for certain DNA uptake sequences (DUS); this is the case for H. influenzae and *Neisseria* spp. This would require a receptor that is specific for each DUS, likely to be confined to a limited range of organisms, as is the case for neisserial ComP (38). In many cases, current knowledge of the atomic details of each step in the uptake process is scant. Recent developments in cryoelectron tomography have provided valuable insights into the assembly of the type IV pilus biogenesis system *in vivo*, revealing a complex with components in the inner and outer membranes, connected by a channel which spans the periplasm (22, 53). These reconstructions are, however, at comparatively low resolution; a complete description of the DNA uptake machine will require atomic-level detail, which can only be achieved by higher-resolution structure determination of the component proteins and their complexes.

The current model for DNA uptake therefore requires a receptor which is able to provide the first encounter with the DNA substrate. It is necessary to invoke the existence of such a receptor, as it is the most plausible way by which DNA is guided into and through the PilQ secretin channel, which is thought to be the conduit for DNA passage across the outer membrane (25). Direct evidence for this hypothesis was recently published by Ellison et al., who demonstrated, in Vibrio cholerae, the binding of type IV competence pili to extracellular DNA and pilus retraction transporting the bound substrate to the cell surface (54). It is reasonable to infer, based on current evidence for the requirement for competence pilins, that such specialized receptors are associated with the competence pilus fiber, most likely present as a specialized pilin. The structure of ComZ reveals a larger and more complex competence-associated pilin than those studied to date. ComP from N. meningitidis adopts a type IV pilin structure but has no additional domains (37). Interaction with DNA is through positively charged residues on the surface, and ComP binds DNA with a specificity which reflects the DUS specificity in *Neisseria* spp. (37, 38). It is unclear, however, if this model for a DUS-specific pilin applies outside the *Neisseriae*. In addition, the observation that uptake is not impaired in a *comP* mutant suggests that other components are involved in the initial process of DNA recognition (39). Competence pili have been directly observed in S. pneumoniae (55), and the solution structure of the component pilin, ComGC, was determined by nuclear magnetic resonance (56). ComGC is the major component of the S. pneumoniae competence-associated pilus (57). It has some characteristics in common with T4a pilins, in that it has a hydrophobic N-terminal α-helix and a Glu at position 5 and is processed by the PilD peptidase. In contrast to ComP, DNA does not bind to the ComGC monomer, although it does do so to the assembled, mature competence pilus (57). There are therefore few points of similarity between N. meningitidis ComP and S. pneumoniae ComGC.

It is in this context that we examined the structures and functions of the competence-associated pilins previously identified in T. thermophilus HB27 (35). Mutation of these pilins gives a noncompetent but piliated phenotype, suggesting that PilA1-3 and ComZ combine to form a competence-specific pilus fiber. Of these four genes, ComZ stood out as the largest and had atypical features for a type IVa pilin, with a Gly at position 5, for example. ComZ is not predicted to be a substrate for the PilD peptidase by PilFind (42), although it should be noted that such a prediction is not infallible, and independent experimental evidence would be required to confirm whether or not ComZ is processed in this way. The structure is indicative of a type IV pilin fold, but with closest similarities to the GspK pseudopilin, which forms a heterotrimer proposed to form the tip of a pseudopilus which drives substrate secretion in the type II secretion system (T2SS) (29). This reflects our earlier observation that other T. thermophilus minor pilins are more closely related to T2SS pseudopilins than T4a pilin structures (35). We also show that ComZ binds to another pilin in the locus, PilA2, but not PilA1 or PilA3, suggesting some specificity of interaction, which would be expected if the pilins were incorporated into the fiber in a specific order. We note that ComZ contains a predicted hydrophobic α-helix, running from residues 5 to 27, which could form the basis of interaction with PilA2 in a fashion similar to that of the assembly of the GspK/GspI/GspJ heterotrimer. The dominant structural feature of ComZ, however, is the large β-solenoid domain, which appears to have been inserted into the pilin fold. The result is a large macromolecule, measuring ∼60 Å across, similar to the diameter of type IV pilus fibers from Gram-negative organisms (27). The latter measurement is significant because a competence pilus fiber would need to navigate through the PilQ secretin channel. Recent structures of the T2SS GspD secretin, which is closely related to PilQ, confirm earlier observations that the central secretin pore is approximately 60 Å across, with the potential to widen to accommodate the emerging pilus fiber and therefore, by implication, a tip-located ComZ (58). In contrast, it is difficult to reconcile incorporation of ComZ into the central body of a type IVa pilus fiber. Atomic models for type IV pilus fibers vary in detail—although none are available for T. thermophilus pilus fibers at present—but are based on the association of hydrophobic N-terminal α-helices at the center of the fiber. We find that the size of the β-solenoid domain, and its limited flexibility with respect to the pilin domain, argues against its incorporation into the main competence pilus fiber; steric constraints make this unlikely. We are left with the more plausible option, which is that ComZ is a tip pilin, as is the case for GspK. This suggests that ComZ is incorporated first into the nascent competence fiber as it is being assembled, followed by PilA2. Our model for the ComZ/PilA2 complex underscores this point by locating PilA2 such that its smaller subdomain sits between the ComZ pilin and β-solenoid domains, suggesting that ComZ could effectively cap the end of the competence pilus fiber (Fig. 8).

DNA binding is an unusual function for a β-solenoid structure, usually associated with hydrolytic enzyme activity (e.g., K5 lyase tailspike protein [59]). An examination of surface electrostatics of the β-solenoid domain did not suggest any characteristic positive charge patches, which are often associated with DNA binding (Fig. S7), but this does not necessarily preclude a DNA binding function. We obtained direct evidence for DNA binding to ComZ by developing a method based on protection of lysine residues against reductive dimethylation. Our resulting structural model suggests a side-on association of DNA with the end of the pilus fiber (Fig. 8). *In vivo*, association of DNA with the competence pilus could well involve other competence pilins and will require further study of the assembly of the fiber.

10.1128/mBio.00614-19.7 FIG S7 Electrostatic surface of ComZ (chain A) computed using CCP4MG (72). Download FIG S7, DOCX file, 0.9 MB. Copyright © 2019 Salleh et al. 2019 Salleh et al. This content is distributed under the terms of the Creative Commons Attribution 4.0 International license.

**FIG 9:**
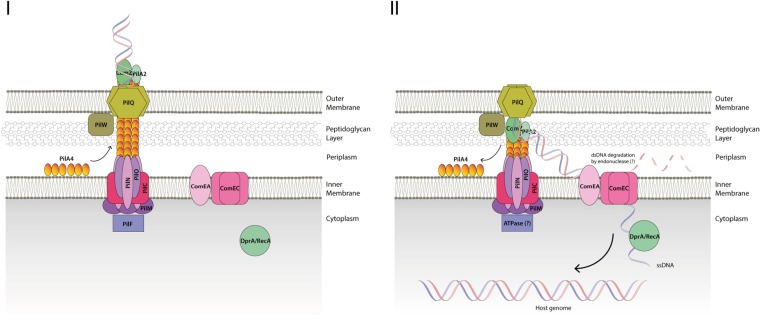
Schematic model for DNA uptake in T. thermophilus. In stage I, the pilin PilA4 (17) is assembled into a competence pilus fiber by the inner membrane complex PilMNO and PilC (14, 73). Elongation of the pilus is powered by the hexameric AAA-ATPase PilF, which provides energy for the assembly via ATP hydrolysis (13, 74). In stage II, double-stranded DNA is bound by the pilus tip-associated DNA receptor ComZ/PilA2 and transported into the periplasm through the secretin pore PilQ (24). A competence-associated retraction ATPase has not been identified and may not, in any case, be required. Once in the periplasm, double-stranded DNA binds to ComEA (6), one strand is degraded by an unidentified endonuclease, and the remaining DNA strand is translocated across the inner membrane through ComEC (20). In the cytoplasm, DNA is used as a source for intracellular metabolism or recombined with the host chromosomal genome (75).

Figure 9 summarizes our proposed model for the role of ComZ in the DNA uptake into T. thermophilus by NT. ComZ is located at the tip of a competence pilus, supported in position by the minor pilin PilA2. It acts as the initial receptor for DNA binding outside the cell; the assembled pilus is retracted to bring the DNA substrate into the periplasm and into close proximity with ComEA and ComEC. DNA is then transferred to ComEA, which is inner membrane associated, and probably works in concert with the ComEC channel. The role of DNA binding to ComEA has been highlighted by the recent observations of Hepp and Maier in *Neisseria* (39). Their model suggests that ComEA binding, in a ratchet-type model, drives DNA uptake. This hypothesis requires that ComEA binds with higher affinity than any DNA receptors or components, which function earlier in the process. This could include a protein with a function similar to that of ComZ and perhaps also the PilQ secretin; indeed, there is evidence that N. meningitidis PilQ binds DNA (25).

The general applicability of our conclusions on the function of ComZ lie in the role of a tip-located pilin with a specific DNA-binding function. This is a feature which could be replicated in other species, even if the structural details are different. Within Gram-negative organisms, NT uptake systems are diverse. Although they have some common constituents, such as the ComA and ComE proteins, other components vary. One reason may be that some organisms exhibit DNA sequence specificity through DUSs, whereas others do not. In addition, regulation differs between different organisms (21). Nevertheless, the structure and properties of ComZ point to the involvement of a specialized, tip-associated T4a pilin for initial DNA recognition, which could have general applicability to other NT systems.

## MATERIALS AND METHODS

### Cloning, expression, and purification of ComZ and PilA1-3.

The *comZ* gene (omitting the region coding for the signal peptidase sequence and hydrophobic part of the N-terminal helix) was amplified by PCR using primers CTTCACCATGGCCATAGAGCTCTGGACCACCCGCAACGAC and CGGTGTGACTCGAGGCGGCGCTCATAGGAGAGCACCTG and T. thermophilus HB27 genomic DNA as the template. The amplified gene and the pET-22b vector (Novagen) were digested with restriction enzymes NcoI and XhoI, purified, and ligated. The *comZ*-22b construct coded for the ComZ protein (residues 31 to 554) with the PelB leader sequence at the N terminus and hexahistidine tag at the C terminus. Synthetic constructs corresponding to the soluble domains PilA1 (residues 30 to 156), PilA2 (residues 33 to 193), and PilA3 (residues 33 to 233) were designed, optimized for expression in E. coli, and synthesized (GeneArt) with a Strep-tag II prior to subcloning into pET-22b vector (BamHI and XhoI sites), which includes the PelB leader at the N terminus. Recombinant plasmids were transformed into T7 Express cells (New England Biolabs [NEB]) for ComZ expression or Lemo21(DE3) cells (NEB) for minor prepilin expression and grown on Luria-Bertani (LB) plates with 100 μg/ml ampicillin (T7 Express) or 100 μg/ml ampicillin and 30 μg/ml chloramphenicol (Lemo21 cells) at 37°C for 14 h. Several colonies were inoculated into 50 ml of starter culture (LB medium with antibiotics) and grown until the optical density at 600 nm (OD_600_) reached 0.6 to 0.8. Cells were subsequently grown in Terrific broth (TB) medium supplemented with antibiotics and were induced with 0.4 mM isopropyl-β-d-thiogalactopyranoside (IPTG) when the OD_600_ reached 0.8. Cells were left shaking at 16°C for 16 h to allow protein expression before being harvested by centrifugation at 8,000 rpm (SLA3000; Sorvall) for 30 min at 4°C. Cell pellets were resuspended in buffer A (25 mM Tris-HCl, pH 8.5, 100 mM NaCl for ComZ and 25 mM Tris-HCl, pH 8.0, 200 mM NaCl for prepilins) supplemented with protease inhibitor cocktail (Roche) and DNase I (5 μg/ml). Cells were disrupted by sonication (Sonopuls; Bandelin) for 7 min at 30% power. Debris and unbroken cells were removed by centrifugation at 18,000 rpm (F21; Sorvail) for 45 min at 4°C. For ComZ, purification was carried out by using a HisTrap HP column (GE Healthcare), followed by an elution step using buffer A plus increasing concentrations of imidazole (up to 500 mM). The purification of Strep-tag II-tagged pilins was carried out on a StrepTrap HP column (GE Healthcare). The streptavidin column was first equilibrated with 5 column volumes (CV) of water and 5 CV of buffer A. Crude cell lysates were applied to the column, which was washed with 6 CV of buffer A, followed by elution with 6 CV of buffer A plus 5% glycerol and 2.5 mM desthiobiotin. Collected fractions from cell lysates, wash, and elution buffer were analyzed by SDS-PAGE. A final purification step by SEC was carried out using a HiLoad 16/600 Superdex 75 PG column (GE Healthcare) in 25 mM Tris-HCl, pH 8.0, 200 mM NaCl, 5% glycerol at 25°C.

### Protein crystallization, data collection, and structural analysis.

Purified ComZ (8 to 10 mg/ml) was crystallized by sitting drop vapor diffusion. Equal volumes (200 nl) of protein and a reservoir solution containing 0.2 M potassium thiocyanate, 0.1 M Bis-Tris propane, pH 6.5, and 20% (wt/vol) polyethylene glycol (PEG) 3350 were mixed and incubated at 293K. For phasing, crystals were soaked with reservoir solution supplemented with 10 mM potassium tetrachloroplatinate(II) (K_2_PtCl_4_) for 2 to 3 h. Crystals were cryoprotected with reservoir solution supplemented with 20% glycerol and flash-cooled in liquid nitrogen. The native ComZ data set was processed using the xia2 (60) automated pipeline implementing XDS (61), XSCALE, and AIMLESS (62) to a resolution of 2.72 Å. For the Pt derivative, as the anomalous signal was weak, the XDS integrated data from four ComZ crystals (treated with K_2_PtCl_4_) were manually scaled and merged using AIMLESS, as implemented in the CCP4 suite (63), to a resolution of 3.5 Å. This gave an anomalous multiplicity of 33 and midslope anomalous normal probability of 1.19. Automated substructure identification, calculation of phases, density modification, and low-resolution preliminary model building were carried out using the AutoSol wizard in Phenix (64). Thirteen Pt sites were identified and produced an interpretable electron density map. At this stage, it was clear that there are three molecules in the asymmetric unit and the presence of the β-solenoid domain. Manual model building was carried out to assign as many residues as possible to regions of interpretable electron density for one of the chains. This chain was then used as a search model for molecular replacement to extend the phases to the native data set using Phaser (65). The model was built using the AutoBuild wizard in Phenix (64) and completed using iterative rounds of manual model building using Coot (66) and refinement using phenix.refine (64). The structure was analyzed using the PDB_REDO server (67) and validated using MolProbity (68). X-ray data collection and refinement statistics are presented in Table S1 in the supplemental material.

Crystals of PilA2 were obtained using MRC 2-well plates containing 200 nl of protein (13 mg/ml) and 200 nl of 0.2 M ammonium nitrate, 0.1 M Bis-Tris propane, pH 8.5, and 18% (vol/vol) PEG Smear High using a Mosquito robot (TTP Labtech). For phasing, crystals were soaked with the reservoir solution, supplemented with solid potassium iodide (KI) and 15% PEG 200 for 5 min. Crystals were cryoprotected with the reservoir solution, supplemented with 20% glycerol, and then flash-cooled in liquid nitrogen. The native data set was processed using xia2 (69), the automated pipeline implementing XDS (61), fast DP, and autoPROC 1.0.5. For the iodide derivative, the XDS integrated data from PilA2 crystals (treated with KI) were manually scaled and merged using AIMLESS (62), as implemented in the CCP4 suite (63), to a resolution of 2.81 Å. This gave an anomalous multiplicity of 6.3 and a midslope anomalous normal probability of 1.103. The model was built using the AutoBuild wizard in Phenix (64) and completed using iterative rounds of the manual model building using Coot (66) and refinement using refmac (70). X-ray data collection and refinement statistics are presented in Table S2.

### Biophysical binding measurements.

For studies of the interaction of ComZ with PilA1-3, analysis was carried out on a HiLoad 16/600 Superdex 200 PG column (GE Healthcare). Prior to the chromatography, the target proteins were incubated together in 25 mM Tris-HCl, pH 8.0, 200 mM NaCl, 5% glycerol at 4°C for 30 min. Affinity tag binding assays used the principle that both ComZ and pilin proteins were expressed with different tags. The ComZ reading frame encoded a C-terminal hexahistidine tag, whereas PilA1, PilA2, and PilA3 incorporated a C-terminal Strep-tag II (WSHPQFEK). A HisTrap HP column was used to study the ComZ-PilA2-PilA3 interaction, and a StrepTrap HP column was used for interaction analysis of ComZ-PilA2 and ComZ-PilA3. Three mg of ComZ and 2 mg of each pilin were loaded in each case. Washing and elution from each affinity column was carried out under the same conditions as those used for affinity chromatography purification in the protein purification protocols described above.

ITC was performed on a MicroCal PEAQ-ITC instrument (Malvern) by titrating 20 2-μl aliquots of PilA2 (314 μM) into the sample cell containing 300 μl of ComZ (33 μM), with rapid stirring at 25°C. Thermodynamic parameters, i.e., binding constant (*K_d_*), stoichiometry (*n*), enthalpy (Δ*H*), and entropy (Δ*S*), were calculated using the manufacturer’s software by measuring heat absorbed or released (71).

Electrophoretic mobility shift assays (EMSA) were carried out by mixing ComZ and linearized DNA plasmid (pET-22b predigested with BamHI), incubation at 4°C for 1 h, and separation by electrophoresis on a 1.5% agarose gel, supplemented with SafeView nucleic acid stain (NBS Biologicals). Increasing concentrations of ComZ (0 μM to 44 μM) were added to a fixed quantity of linearized pET-22b (200 ng) in 25 mM Tris-HCl, pH 8.5, 100 mM NaCl in a final volume of 10 μl. After electrophoresis at a constant voltage (110 V) for 30 min, the gel was visualized and photographed under UV light using a UVpro transilluminator (UVItec). For the DNase protection assay, linearized pET-22b (200 ng) was added to 8.7 μM ComZ in 25 mM Tris-HCl, pH 8.5, 100 mM NaCl in a final volume of 10 μl and incubated at 55°C for 15 min. Samples were cooled to 37°C, DNase I was added to the specified concentration, and samples were incubated for a further 30 min at 37°C prior to separation by electrophoresis.

### Reductive dimethylation protection.

Protection from reductive dimethylation was used to map surface-exposed lysine residues on ComZ, which bind DNA. The protocol was adapted from a method described by Rayment (49). Purified ComZ was exchanged into 50 mM HEPES, pH 7.5, using a Pierce polyacrylamide spin desalting column (7-kDa molecular weight cutoff; Thermo Fisher). Reductive dimethylation was carried out by addition of 20 mM borane-dimethylamine (BDC; Sigma-Aldrich) and 38 mM formaldehyde (final concentrations) in 500 μl of 70 μM ComZ in the presence or absence of 1 μg pET22b linearized by BamHI digestion. The solution was incubated at 0°C for 2 h before addition of a further 20 mM BDC–38 mM formaldehyde and incubation at the same temperature for a further 2 h. Following a third addition of 10 mM BDC, the solution was incubated for a further 14 h at 0°C. The reaction was quenched by addition of glycine to a final concentration of 124 mM, incubated for 1 h at 0°C, and dialyzed into 50 mM HEPES, pH 7.5, for 4 h at 4°C. A further 14-h dialysis was performed against 50 mM HEPES, pH 7.5, 2 mM dithiothreitol (DTT) at the same temperature to reverse any modifications of cysteine and methionine residues.

### Mass spectrometry.

For trypsin digestion, 500 μl of 10.5 μM purified ComZ was incubated at 90°C for 1 h in 20 mM ammonium bicarbonate and 10 mM DTT and cooled to 22°C, and iodoacetamide was added to a final concentration of 10 mM. The reaction mix was incubated in the dark at 22°C for 45 min before addition of trypsin at a mass ratio of 1:75 and incubated for 14 h at 37°C. Samples were desalted by reverse-phase, solid-phase extraction using Oligo R3 beads (Thermo Scientific) with elution in 40% acetonitrile in 0.1% formic acid, followed by evaporation via vacuum centrifugation and subsequent resuspension in 10 μl of 5% acetonitrile in 0.1% formic acid. Samples were analyzed by LC-tandem MS (LC-MS/MS) using an UltiMate 3000 rapid separation LC (RSLC; Dionex Corporation, Sunnyvale, CA) coupled to an Orbitrap Elite (Thermo Fisher Scientific, Waltham, MA) mass spectrometer. Peptide mixtures were separated using a gradient from 92% A (0.1% FA in water) and 8% B (0.1% FA in acetonitrile) to 33% B in 44 min at 300 nl min^−1^, using a 75-mm by 250-μm-inner-diameter 1.7 μM CSH C_18_ analytical column (Waters). Peptides were selected for fragmentation automatically by data-dependent analysis. The data produced were then analyzed using Progenesis QI for Proteomics software (Waters) to align and quantify the peptide signals, which were then identified using Mascot software (Matrix Sciences) from the Swiss-Prot database of protein sequences (European Bioinformatics Institute) from January 2018, using oxidation on methionines and dimethylation on lysines as variable modifications. The signal intensities of peptides that were identified from ComZ were compared to determine the level of protection from dimethylation.

### Data availability.

Structure factors and atomic coordinates for ComZ and PilA2 have been deposited in the Protein Data Bank with accession codes 6QVI and 6QVF, respectively.
