# Supplementary material for: Structure and Properties of a Natural Competence-Associated Pilin Suggest a Unique Pilus Tip-Associated DNA Receptor
Source: mBio. 2019 Jun 11;10(3):e00614-19. doi: 10.1128/mBio.00614-19 (PMC6561018; doi:10.1128/mBio.00614-19)
Supplement: TABLE S1 [file mBio.00614-19-st001.docx]

# Table S1 X-ray data collection and refinement statistics for ComZ

|  | **ComZ (native)** | **ComZ (Pt derivative)** |
| --- | --- | --- |
| **Data collection**  Space group  Number of crystals  Unit cell dimensions  X-ray source  Wavelength (Å)  Resolution range (Å)  Completeness (%) ^b^  Multiplicity  I/σ I  R_merge_^c^  R_meas_^d^  R_pim_  Total observations  Total unique  Anomalous completeness  Anomalous multiplicity  Mid-slope of anomalous normal probability    **Refinement**  Resolution range (Å)  R-work  R-free  RMS (bonds)  RMS (angles)  Average B-factor (Å^2^)  **Ramachandran plot (%)**  Favored  Allowed  Outliers | *P3_1_21*  1  a=b=121.86 Å, c=212.35 Å; α=β=90°, γ=120°  DLS I02^a^  1.0  58.79-2.72 (2.79-2.72)  99.9 (99.9)  5.8 (6.1)  18.1 (2.0)  0.058 (1.065)  0.070 (1.280)  0.029 (0.515)  286,903 (21,967)  49,699 (3,613)  58.79-2.72 (2.78-2.72)  0.205 (0.362)  0.261 (0.436)  0.0116  1.55  96.0  93.89  6.11  0 | *P3_1_21*  4  a=b=126.08 Å, c=216.50 Å; α=β=90°, γ=120°  DLS I04-1  0.9282  109.09-3.5 (3.74-3.50)  99.9 (99.7)  63.0 (34.3)  13.8 (2.4)  0.388 (2.101)  0.394 (2.157)  0.067 (0.467)  1,625,376 (156,751)  25,782 (4,568)  99.9 (99.7)  33.1 (17.1)  1.194 |

^a^ Diamond Light Source

^b^ values in parentheses refer to the outer resolution shell

^c^ *R_merge_*= $\frac{\sum_{hkl} \sum_{j} |I_{hkl,j}-<I_{hkl}>|}{\sum_{hkl} \sum_{j} I_{hkl,j}}$

^d^ *R_meas._* =$\frac{\sum_{hkl} \sqrt{\frac{n}{n-1}}\sum_{j=1}^{n} |I_{hkl,j}-<I_{hkl}>|}{\sum_{hkl} \sum_{j} I_{hkl,j}}$

^e^ *R_p.i.m._* =$\frac{\sum_{hkl} \sqrt{\frac{1}{n-1}}\sum_{j=1}^{n} |I_{hkl,j}-<I_{hkl}>|}{\sum_{hkl} \sum_{j} I_{hkl,j}}$
