# Supplementary material for: Structure and Properties of a Natural Competence-Associated Pilin Suggest a Unique Pilus Tip-Associated DNA Receptor
Source: mBio. 2019 Jun 11;10(3):e00614-19. doi: 10.1128/mBio.00614-19 (PMC6561018; doi:10.1128/mBio.00614-19)
Supplement: FIG S3 [file mBio.00614-19-sf003.docx]

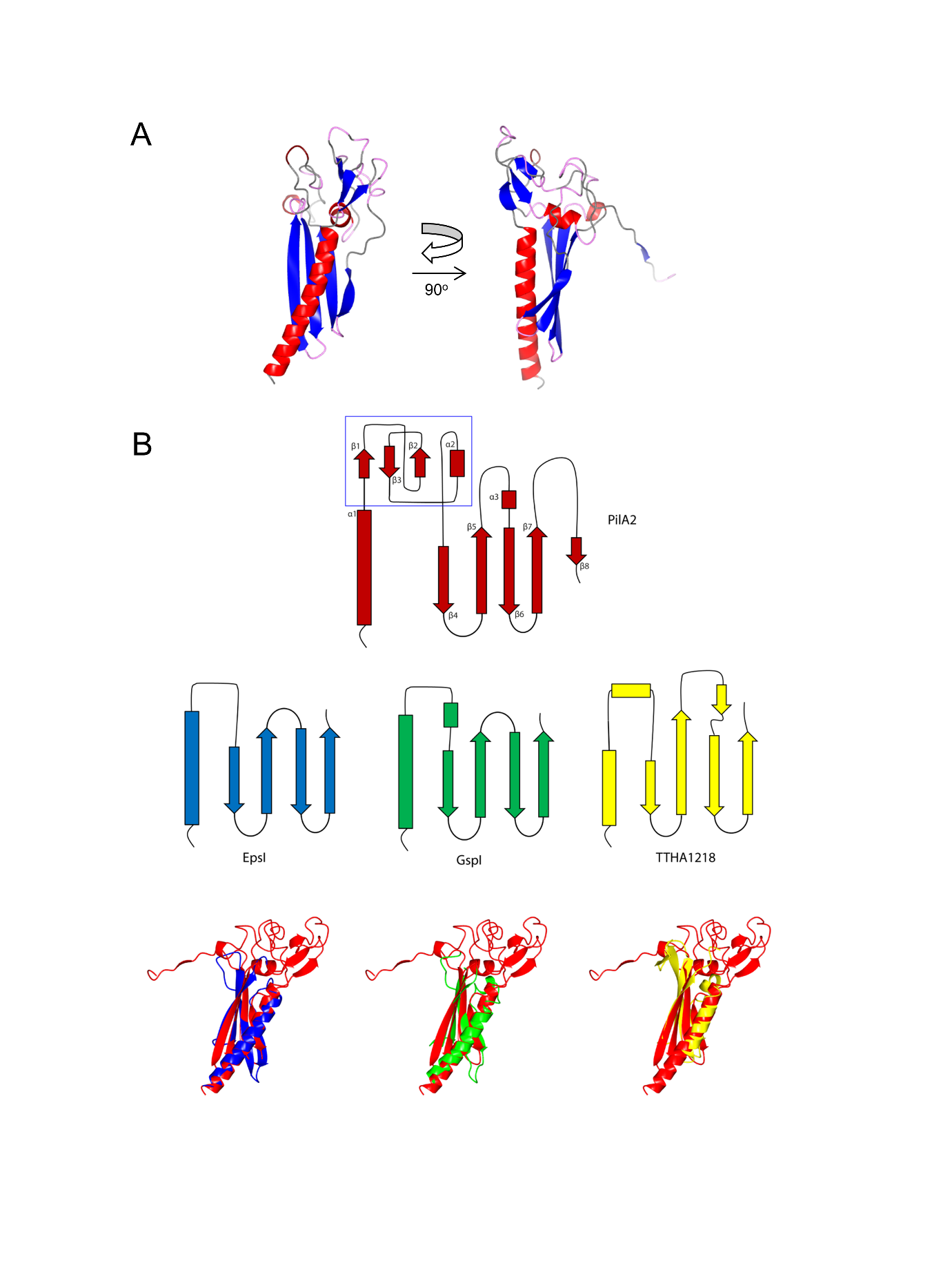


**Figure S3 Structure of the competence-associated type IV pilin PilA2.** (A) Two orthogonal ribbon plot views of PilA2, colored by secondary structure. (B) Upper panel, topology diagram for PilA2. The additional sub-domain is indicated by the blue box; middle panel, topology diagrams for EpsI from *V. cholerae*, GspI from *E. coli* and TTHA1218 from *T. thermophilus*; lower panel, superposition of PilA2 structure (red) with EpsI (blue), GspI (green) and TTHA1218 (yellow). See main text for other details.
