# Supplementary material for: Structure and Properties of a Natural Competence-Associated Pilin Suggest a Unique Pilus Tip-Associated DNA Receptor
Source: mBio. 2019 Jun 11;10(3):e00614-19. doi: 10.1128/mBio.00614-19 (PMC6561018; doi:10.1128/mBio.00614-19)
Supplement: TABLE S2 [file mBio.00614-19-st002.docx]

# Table S2 X-ray data collection and refinement statistics for PilA2

|  | **PilA2 (native)** | **PilA2 (KI derivative)** |
| --- | --- | --- |
| **Data collection**  Space group  Number of crystals  Unit cell dimensions    X-ray source  Wavelength (Å)  Resolution range (Å)  Completeness (%)^b^  Multiplicity  I/σ I  R_merge_  R_meas_  R_pim_  Total observations  Total unique  Anomalous completeness  Anomalous multiplicity  Mid-slope of anomalous  normal probability    **Refinement**  Resolution range (Å)  R-work  R-free  RMS (bonds)  RMS (angles)  Average B-factor (Å^2^)  **Ramachandran plot (%)**  Favored  Allowed  Outliers | *P2_1_*  1  a=50.59 Å, b=137.82 Å, c=59.74 Å; α=γ=90°, β=98.18°  DLS I04^a^  0.98  54.34-1.39 (1.44-1.39)  86.5 (59.3)  3.4 (2.9)  8.64 (0.65)  0.059 (1.306)  0.070 (1.608)  0.038 (0.923)  519,353 (39,904)  154,421 (9,515)  69.00-1.44  0.151  0.189  0.011  1.43  25.10  99.41  0.59  0.00 | *P2_1_*  1  a=50.81 Å, b=137.62 Å, c=59.26 Å; α=γ=90°, β=97.73°  DLS I04  1.5  29.68-2.81 (2.86-2.81)  97.7 (54.6)  12.7 (8.9)  6.5 (2.8)  0.395 (0.492)  0.414 (0.518)  0.121 (0.157)  244,710 (4,583)  19,228 (517)  96.9 (61.2)  6.3 (6.0)  1.103 |

^a^ Diamond Light Source

^b^ values in parentheses refer to the outer resolution shell

^c^ *R_merge_*= $\frac{\sum_{hkl} \sum_{j} |I_{hkl,j}-<I_{hkl}>|}{\sum_{hkl} \sum_{j} I_{hkl,j}}$

^d^ *R_meas._* =$\frac{\sum_{hkl} \sqrt{\frac{n}{n-1}}\sum_{j=1}^{n} |I_{hkl,j}-<I_{hkl}>|}{\sum_{hkl} \sum_{j} I_{hkl,j}}$

^e^ *R_p.i.m._* =$\frac{\sum_{hkl} \sqrt{\frac{1}{n-1}}\sum_{j=1}^{n} |I_{hkl,j}-<I_{hkl}>|}{\sum_{hkl} \sum_{j} I_{hkl,j}}$
