# Supplementary material for: Structure and Properties of a Natural Competence-Associated Pilin Suggest a Unique Pilus Tip-Associated DNA Receptor
Source: mBio. 2019 Jun 11;10(3):e00614-19. doi: 10.1128/mBio.00614-19 (PMC6561018; doi:10.1128/mBio.00614-19)
Supplement: FIG S4 [file mBio.00614-19-sf004.docx]

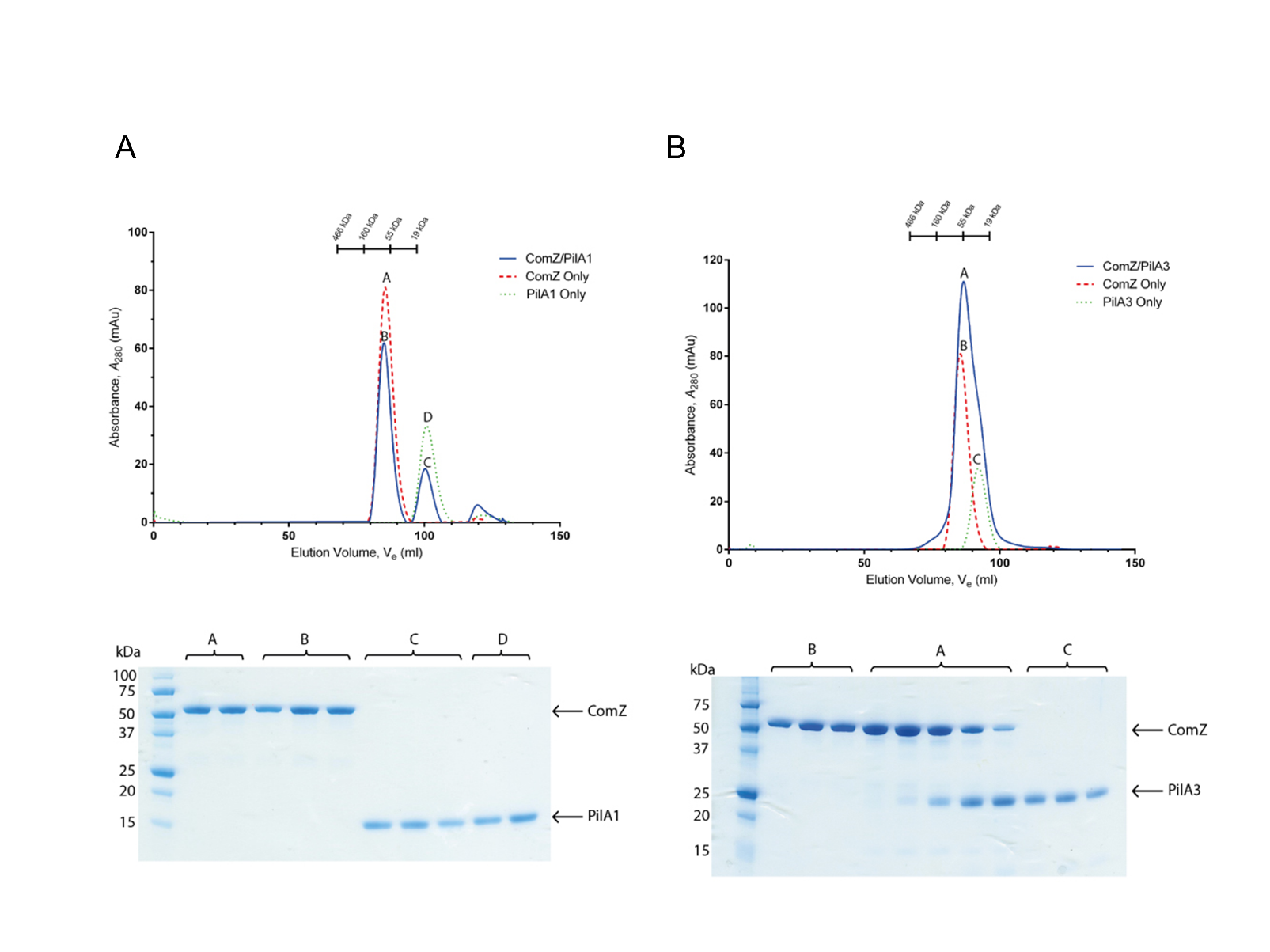


**Figure S4.** **Size exclusion chromatography analysis of ComZ interaction with PilA1 and PilA3.** (A) ComZ and PilA1. Upper panel: elution profiles (absorption at 280 nm) of ComZ/PilA1 mixture (profile B and C), ComZ alone (profile A) and PilA1 alone (profile D). Separation was carried out on a HiLoad 16/600 Superdex 200 PG column (GE Healthcare), flow rate 1 ml/min in buffer 25mM Tris-HCl pH8.0, 200 mM NaCl, 5% glycerol. Prior to SEC separation, the proteins were incubated in the buffer at 4° C for 30 minutes. 0.036 µmol of ComZ and 0.027 µmol of PilA1 were loaded. Lower panel: SDS-PAGE of eluted peaks from each SEC run. (B) ComZ and PilA3. Upper panel: elution profiles (absorption at 280 nm) of ComZ/PilA3 mixture (profile A), ComZ alone (profile B) and PilA3 alone (profile C). SEC separation was carried out as for panel (A).
