# Supplementary material for: Structure and Properties of a Natural Competence-Associated Pilin Suggest a Unique Pilus Tip-Associated DNA Receptor
Source: mBio. 2019 Jun 11;10(3):e00614-19. doi: 10.1128/mBio.00614-19 (PMC6561018; doi:10.1128/mBio.00614-19)
Supplement: FIG S6 [file mBio.00614-19-sf006.docx]

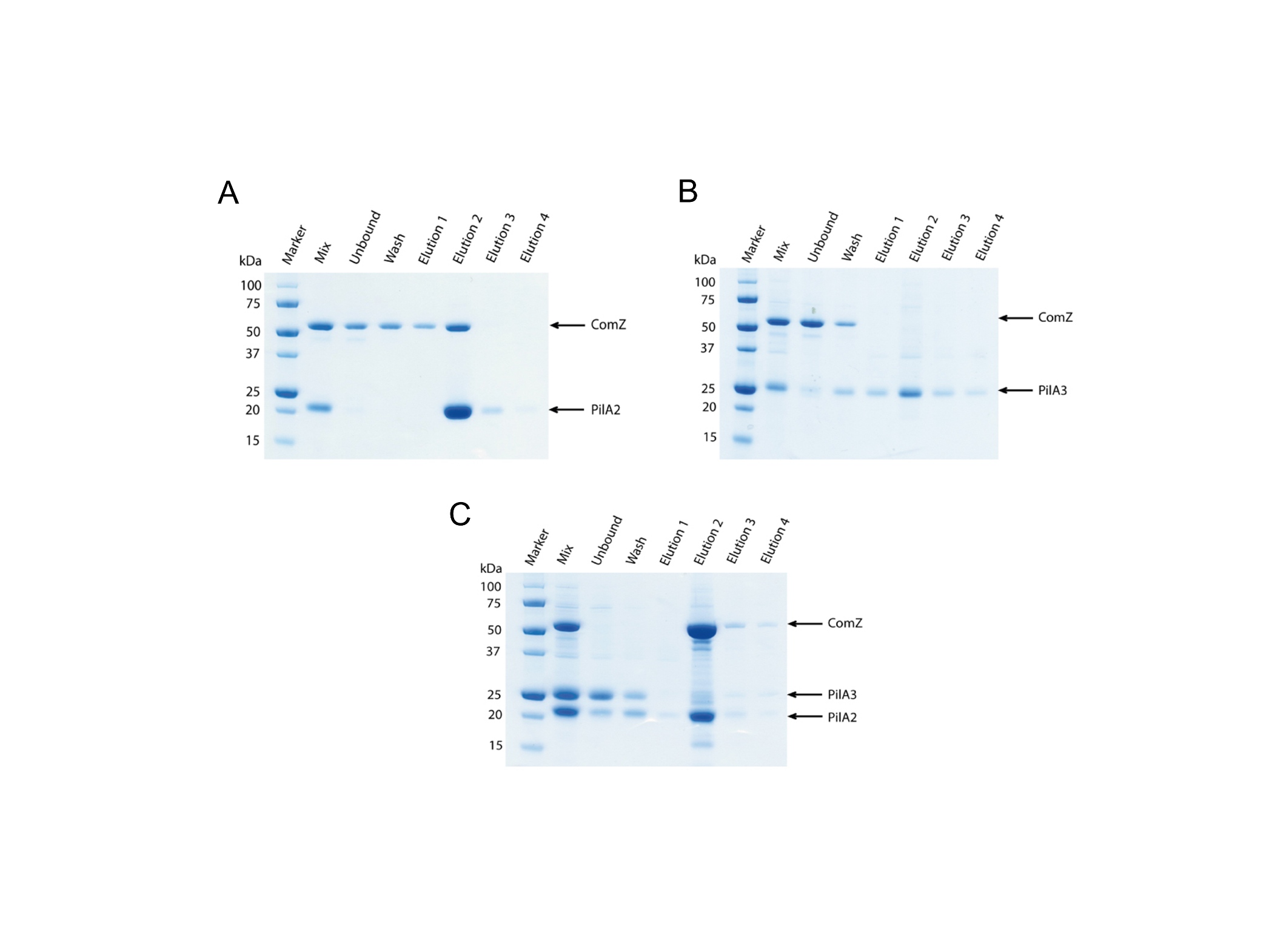


**Figure S6 ComZ affinity binding assays.** (A) ComZ/PilA2: both proteins were incubated in 25mM Tris-HCl pH8.0, 200 mM NaCl, 5% glycerol at 4 °C for 30 minutes, applied to a Streptavidin column and eluted proteins examined by SDS-PAGE. 3 mg of ComZ and 2 mg of PilA2 were loaded. (B) ComZ/PilA3: both proteins were incubated together, applied to a Streptavidin column and eluted proteins examined by SDS-PAGE. 3 mg of ComZ and 2 mg of PilA3 were loaded. Buffer and temperature were as for panel (A). (C) ComZ/PilA2/PilA3: both proteins were incubated together, applied to a Ni affinity column and eluted proteins examined by SDS-PAGE. Buffer and temperature were as for panel (A).
