# Supplementary material for: Structure and Properties of a Natural Competence-Associated Pilin Suggest a Unique Pilus Tip-Associated DNA Receptor
Source: mBio. 2019 Jun 11;10(3):e00614-19. doi: 10.1128/mBio.00614-19 (PMC6561018; doi:10.1128/mBio.00614-19)
Supplement: FIG S7 [file mBio.00614-19-sf007.docx]

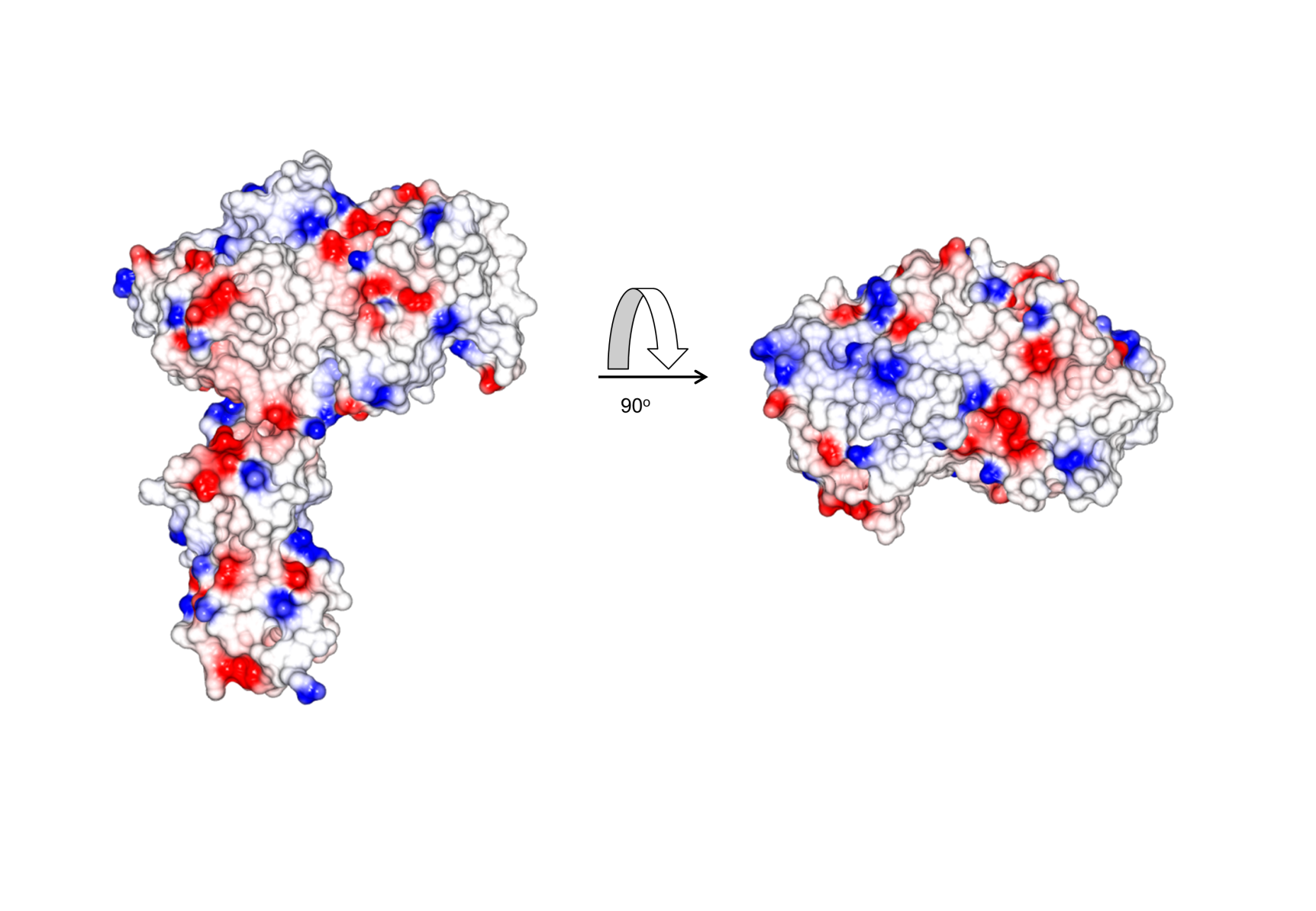


**Figure S7.** Electrostatic surface of ComZ (chain A) computed using CCP4MG[^1^](#_ENREF_1).

**References**

1. Potterton L.*, et al.* Developments in the CCP4 molecular-graphics project. *Acta Crystallogr Sect D Biol Crystallogr* **60**, 2288-2294 (2004).
